# Dynamic coupling of plasmonic resonators

**DOI:** 10.1038/srep21989

**Published:** 2016-02-25

**Authors:** Suyeon Lee, Q-Han Park

**Affiliations:** 1 Department of Physics, Korea University, Seoul 136-701, Korea

## Abstract

We clarify the nature of dynamic coupling in plasmonic resonators and determine the dynamic coupling coefficient using a simple analytic model. We show that plasmonic resonators, such as subwavelength holes in a metal film which can be treated as bound charge oscillators, couple to each other through the retarded interaction of oscillating screened charges. Our dynamic coupling model offers, for the first time, a quantitative analytic description of the fundamental symmetric and anti-symmetric modes of coupled resonators which agrees with experimental results. Our model also reveals that plasmonic electromagnetically induced transparency arises in any coupled resonators of slightly unequal lengths, as confirmed by a rigorous numerical calculation and experiments.

Plasmonic nano resonators formed by various metallic nanostructures are at the core of plasmonics and metamaterial researches. Novel features arise when resonators are coupled to each other, including cases such as plasmonic dimers^1^,^2^,^3^, plasmon induced transparency^4^,^5^,^6^,^7^,^8^,^9^,^10^,^11^, directional optical antenna^12^,^13^,^14^ and high refractive index metamaterials^15^,^16^. The interaction of localized plasmons in nanoparticle dimers has been explained in terms of a simple dipolar interaction^17^ or plasmon hybridization^1^,^18^, the more general method. Though these approaches provide a reasonable description of coupled nanoparticles, they fail to accommodate the dynamical behaviour of coupled plasmonic resonators with accurate quantitative predictions, one of the most important open questions in plasmonics and metamaterial research. Recently, a single plasmonic resonator has been rigorously identified as a radiating dipole oscillator^19^,^20^. A plasmonic resonator, for instance a half-wavelength rectangular hole in a metal film, can be identified as a bound charge oscillator, and, accordingly, the scattering of light by a resonator can be quantitatively described in terms of the scattering spectrum of a bound charge oscillator^20^,^21^. This realisation raises the possibility of rigorously describing the interaction between plasmonic resonators in terms of bound charge oscillators.

In this Letter, we clarify the nature of dynamic coupling in plasmonic resonators by showing that plasmonic resonators couple to each other through the retarded interaction of bound charge oscillators. We determine the dynamic coupling of resonators explicitly from their retarded interaction and screened charges, allowing us to develop quantitative descriptions of the fundamental symmetric and anti-symmetric modes of coupled resonators in good agreement with actual experiments and exact numerical results. Importantly, our dynamically coupled oscillator model predicts the presence of the plasmonic electromagnetically induced transparency (EIT) in any coupled resonators of unequal lengths. Our model provides an analytic and for the first time quantitative description of EIT in plasmonic resonators. We show that the EIT of coupled oscillators arises from the destructive interference of two fundamental modes with distinct phase properties and that, as we confirm experimentally, the same feature arises in coupled plasmonic resonators. Our dynamic coupling approach is not restricted to the rectangular hole-type resonators considered in this paper. It can be readily applied to interacting plasmonic resonators in general and various building blocks of metamaterials.

## Results

### Oscillator picture of coupled resonators

We first recall that a single plasmonic resonator can be identified as a radiating bound charge dipole oscillator^20^. Consider for example a narrow rectangular hole of size *a* × *b* in a thin metal film. When light is incident upon the rectangular hole with a polarization perpendicular to its long side, the total cross section of the free standing rectangular hole is given by the normalized energy flow through it,


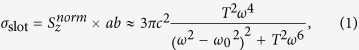


where *c* is the speed of light, 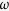 is the angular frequency of the incident wave and 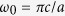. *T* is the dispersive time parameter,


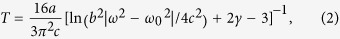


with the Euler’s constant 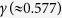. It was noted that a similar scattering property arises in a bound charge oscillator obeying the equation of motion,


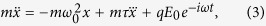


where *x*(*t*) is the displacement from the equilibrium position of a particle of mass *m* and charge *q*. Terms in the right side of Eq. (3) represent the restoring force, the radiative reaction force and the external force, respectively. The radiative reaction force, also known as the Abraham-Lorentz force, is responsible for the radiative energy loss characterized by the time constant 
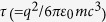
. The total cross section of a bound charge oscillator 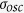 is defined as the ratio of the time-averaged radiated power to the incident intensity. Using the Larmor formula, 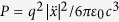, we find


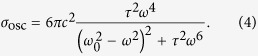


Comparing Eqs. (1) and (4) reveals that the scattering cross section is identical, after allowing for the 50% reduction in intensity for the slot case which represents only forward scattering. If we choose 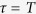, or equivalently introduce a dispersive oscillator mass,


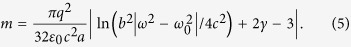


**Figure 1:**
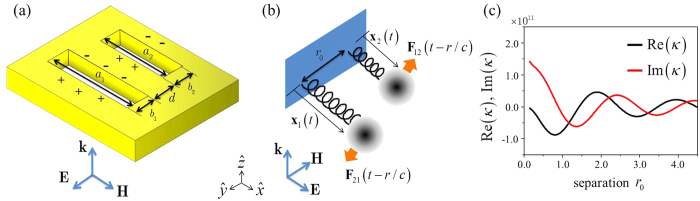
(**a**) Coupled slot resonators made of two rectangular holes of dimensions 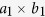 and 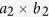 separated by distance 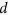 in a perfect electric conductor (PEC) of negligible thickness. Light is incident normally upon the rectangular hole with a polarization perpendicular to its long side. (**b**) Bound charge oscillator model corresponding to coupled slot resonators. Bound charges, separated by distance 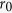 at rest, oscillate along the x-direction only. (**c**) Dynamic coupling constant 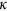 against charge separation.

Now, we consider two interacting plasmonic resonators, two rectangular holes of dimensions 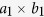 and 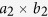 separated by distance *d* as shown in Fig. 1a. Light is incident upon the holes with a polarization perpendicular to the long sides of the rectangles. The oscillator model of a plasmonic resonator suggests that an interaction between resonators can be treated as if it were an interaction between oscillating bound charges and we show that this is indeed the case. Since rectangular holes are complementary structures to rod type resonators, Babinet’s principle indicates that the corresponding charged oscillators can be restricted to movement along the *x*-direction with displacements 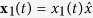 and 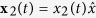 as shown in Fig. 1b. The equations of motion for coupled oscillators are


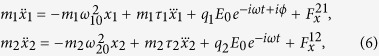


where 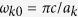 and a phase difference 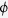 is allowed between the externally applied forces. The term 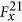 is the *x*-component of the Lorentz force **F**_21_ acting on *q*_*1*_ and likewise for 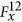 with 1↔2,


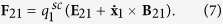


We include the effect of charge screening on the interaction of plasmonic resonators using a screened charge 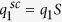 with a screening factor *S* which will be determined later. **E**_21_ and **B**_21_ are the electric and magnetic fields due to charge *q*_*2*_ measured at the retarded time *t*–*r*/*c*,


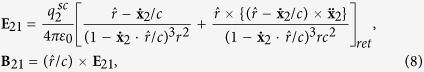


where 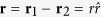 with unit vector 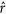 and **r**_**1**_ and **r**_**2**_ are the position vectors of the charges *q*_*1*_ and *q*_*2*_ respectively. We assume that displacements *x*_*1*_(*t*) and *x*_*2*_(*t*) are much smaller than separation *d* so that 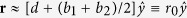. This, together with the harmonic time dependence of *x*_*2*_ with a factor 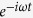, simplifies 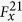 to yield


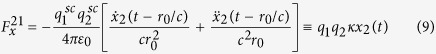


with the dynamic coupling coefficient


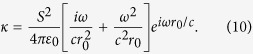


The time-averaged power provided by the external field to charge 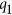 is


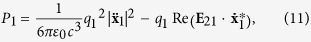


with the equivalent expression for power *P*_*2*_ and charge *q*_*2*_ also holding. Then the total cross section 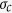 of the coupled oscillator, defined as the ratio of the total power *P*_1_ + *P*_2_ to the incident intensity, becomes


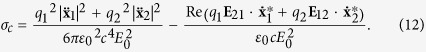


### Determination of dynamic coupling

For a better understanding of coupling and charge screening, we consider the case of two identical oscillators with 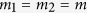 and 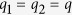. Coupled identical oscillators admit two fundamental modes, symmetric and anti-symmetric. The symmetric mode is driven by external fields without a phase difference (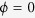) and is characterized by the steady state solution (see Supplementary Information for detailed derivation of Eq (13)–(17), 40, 42, 43, 47)


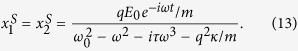


The corresponding total cross section becomes


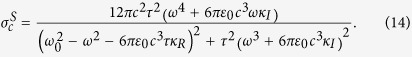


Similarly, the anti-symmetric mode is driven by external fields of phase difference 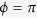 and given by


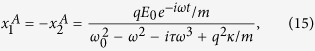


so that the corresponding total cross section becomes


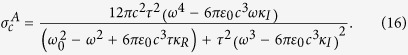


Here, 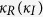 are the real(imaginary) part of the coupling coefficient 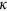 in Eq. (10) which without the factor *S*^2^ becomes singular as the separation *r*_0_ between the two charges becomes zero. In the case of coupled plasmonic resonators of identical dimensions *a* × *b*, as the separation between the two rectangular holes becomes zero, they merge into a single rectangular hole of size *a* × 2*b*. Since the scattering cross section in (4), evaluated near the resonance 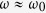, is independent of the short side *b*, at the limit of zero separation we would expect the total scattering cross section of coupled identical resonators to reduce to that of a single resonator. It can be readily shown that this requirement is fulfilled with the choice of the screening factor *S* = *S*_0_ where


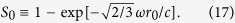


Finally, fixing the dynamic coupling coefficient in (10) with *S* = *S*_0_ as shown in Fig. 1c, we establish the oscillator model for dynamically coupled resonators.

**Figure 2:**
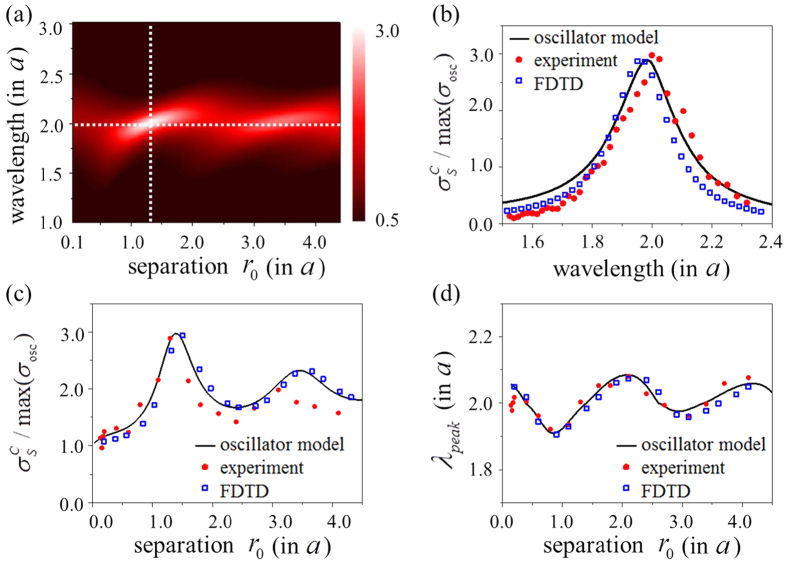
(**a**) Scattering cross section of the oscillator model in symmetric mode plotted against incident wavelength and charge separation. Scattering cross section is normalized by that of single resonator at the resonance, and wavelength and separation are in the unit of the long side of the rectangle 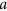. (**b**) Vertical breaks of (**a**) from the oscillator model (thick line) in comparison with the experiment (thin line), and the FDTD calculation (square dot). Separations between oscillators are 1.3a. (**c**) Horizontal break of (**a**) at resonance wavelength (black line) in comparison with the experiment (red dot), and the FDTD calculation (square dot). (**d**) Resonance peak shifts of the cross section in symmetric mode.

To confirm the validity of our model, we compare the total cross sections of coupled oscillators with numerically-calculated and experimentally-measured cross sections of corresponding coupled resonators. The results are shown in Fig. 2 in which the spectrum of the scattering cross section in symmetric mode given by (14) is plotted against resonator separation (the antisymmetric case is given in Supplementary Fig.S1). The scattering cross section is normalized by maximum value of the cross section from single resonator shown in Eq.(4). In Fig. 2b, the vertical breaks in the oscillator scattering cross section represent scattering spectra at the maximum scattering separation. The horizontal break shown in Fig. 2c at *λ* = *λ*_0_ = 2*a* (the black solid line) indicates that the cross section varies from 1 to 3 and converges to 2 in an oscillatory manner as the separation distance increases. This is in accordance with the expectation that the effect of coupling diminishes with larger separations. The positions of the spectral peaks in Fig. 2d show both blue and red shifts from the single resonator resonance, *λ* = *λ*_0_ = 2*a*, depending on the degree of separation of the coupled resonators. Remarkably, all these properties predicted by the oscillator model turn out to be in excellent agreement with not only the scattering behaviour of coupled resonators calculated using a rigorous FDTD numerical method but also the experimental data from microwave measurement.

### Plasmonic EIT in asymmetric resonators

One of the most intensively studied properties of coupled resonators is the plasmonic analogue of electromagnetically induced transparency (EIT) which arises from the interference between bright (symmetric) and dark (anti-symmetric) modes. Various coupled resonator systems such as the dipole-quadrupole antenna pair^10^,^11^ have been employed to demonstrate EIT but without analytic quantitative predictions. Here, we show that a simple system of two parallel rectangular holes of unequal size indeed leads to EIT by opening a dip in the scattering cross section. Our oscillator model approach presents an analytic expression for the scattering cross section which also shows reasonable quantitative agreement with experimental results. Consider the two non-identical rectangular holes specified in Fig. 1 where the aspect ratio 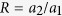 is close to one. The steady state solution of the corresponding oscillator equation is given by (see Supplementary Information for derivation)


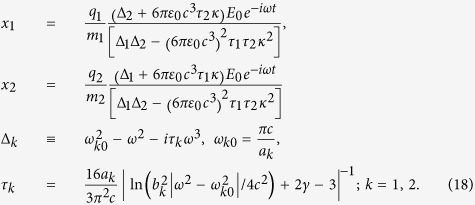


The total scattering cross section is readily obtained from Eq. (12). In order to see the EIT behaviour explicitly, we note that the coupling constant can be approximated to 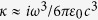 if the separation of the rectangular holes is small. Then, the total scattering cross section simplifies to


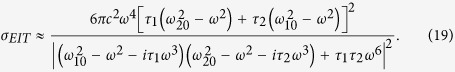


**Figure 3:**
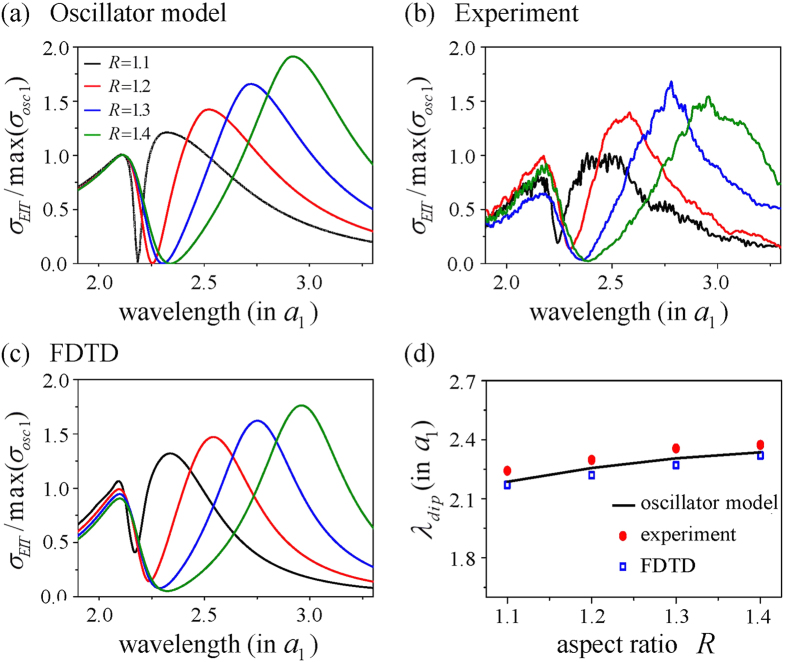
Plasmonic EIT in dynamically coupled resonators. Systems consist of two non-identical rectangular holes of dimensions 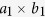 and 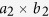 where the aspect ratio 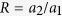 is chosen specifically with values 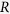
=1.1 (black), 1.2 (red), 1.3 (blue), and 1.4 (green) and 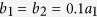. (**a**) Scattering cross section spectra of corresponding oscillator models obtained from Eq. (19). (**b**) Microwave measurement of scattering cross sections of coupled resonators and (**c**) the FDTD numerical results. Scattering cross section is normalized by the cross section of resonant (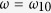) single oscillator of dimension 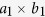 at the resonance, and wavelength is in the unit of 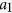. (**d**) Spectral dip positions against the aspect ratio obtained from the oscillator model (black line), the experiment (red circle), and the FDTD calculation (blue square).

**Figure 4:**
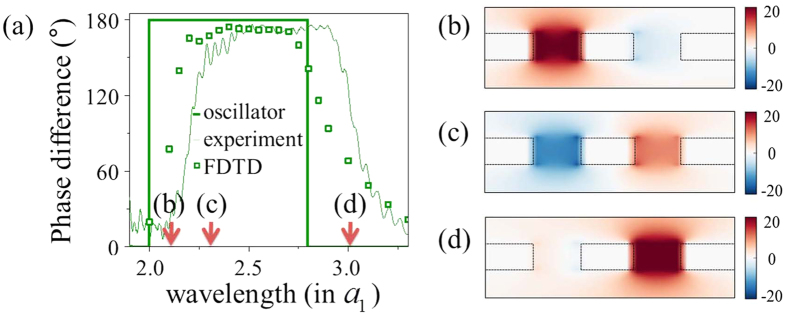
Properties of EIT modes. (**a**) Spectral phase differences in coupled resonators possessing the aspect ratio 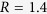 and in the corresponding oscillator. The phase difference of the 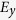-field component has been measured at the center of the two slots separated by distance 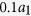 using a hairpin antenna (thin curve). This is compared with the FDTD result (square dot) and the phase difference in two bound charge oscillators (thick curve). (**b**–**d**) 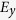 field maps obtained by the FDTD calculation showing the excitation of the dark mode of EIT. Modes at (**b**) the first peak, (**c**) the dip and (**d**) the second peak of EIT spectrum.

Note that 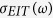 possesses zero, 
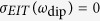
, at


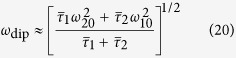


where we approximated 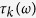 as 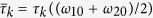. This causes a dip in the broad resonance peak as shown in Fig. 3. Figure 3a shows the cross section spectrum in (19) while Fig. 3b,c show respectively the experimental and the FDTD numerical results for the scattering cross section of two rectangular holes corresponding to four different aspect ratios. Despite the simplicity, our oscillator model prediction manifesting EIT behaviour shows remarkable agreement with both the experimental and rigorous numerical results. Figure 3d compares the dip position predictions of the oscillator model with numerical and experimental results and once again the agreement is good since it is within 2 percent. Figure 4 describes the distinct spectral phase properties of EIT. We compared the phase differences between two oscillators with those between coupled resonators. To show phase difference between slots, we measure near-field of each slot using hairpin antenna. The 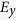-field component has been measured near the edge of slots. The fields resonantly excited inside each of the rectangular holes correspond to the first and the second peaks in Fig. 4b,d. Around the transmission dip, both holes are excited and a 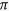-phase difference is expected to arise due to the resonant excitation of a dark mode as shown in Fig 4c,a confirms that this is indeed the case.

## Discussion

In conclusion, we have successfully described the behaviour of interacting plasmonic resonators with a coupled oscillator model which has been derived analytically from the full diffraction theory. The dynamic coupling coefficient of the oscillator model has been determined by the retarded interaction between oscillating screened charges. The oscillator model allowed quantitative predictions about interacting resonators, particularly their EIT behaviour, which agreed with both numerical and experimental results. Our oscillator model approach is not restricted to rectangular-holed plasmonic resonators but can be readily applied to other types of plasmonic resonator such as rod-type nano antennae. Features of the bound charge oscillator model such as radiative damping, retarded interaction, and screening effects are also shared by coupled plasmonic resonators. This suggests that the dynamic coupling approach presented in this paper provides a general analytic framework for coupled resonators.

## Methods

**Figure 5:**
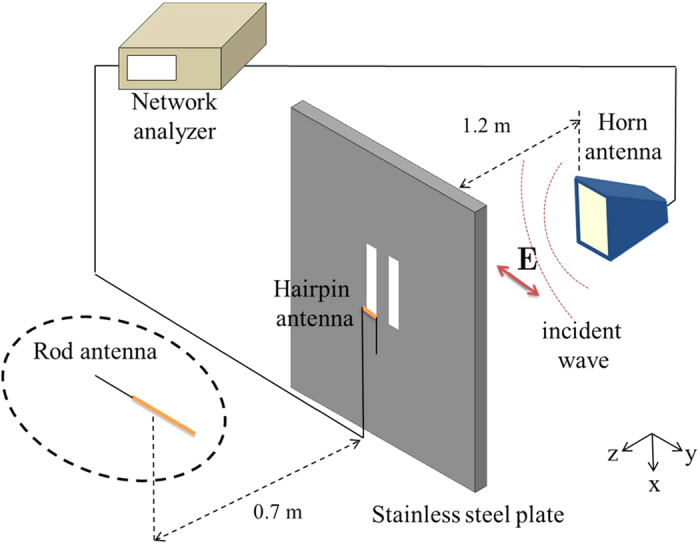
Experimental setup for measuring the microwave coupling of plasmonic resonators. We used the horn antenna as a source of y-polarized electric field, the rod antenna as a receiver for the far field detection, and the hairpin antenna for the near field detection.

Microwave measurements have been performed using the experimental setup in Fig. 5. Slots are perforated on a stainless steel, which exhibits a nearly ideal metal behavior at micro-frequency of 2.6–3.9 GHz. A Hewlett-Packard 8719C network analyzer and a SGH260 standard gain horn antenna were used to generate the y-polarized electric field. To reduce the background noise, we surrounded the experimental setup using KSS-12 pyramidal absorbers. To measure the far and near components of electric field, we used a rod antenna made of a LMR-400 coaxial cable, and a hairpin antenna made of a RG-405 coaxial cable. Since the forward scattering amplitude is proportional to the total cross section according to the optical theorem, we have measured the forward scattering amplitude from a double slot and normalized it by the maximum value of the forward scattering amplitude from a single slot. To measure the phase difference between slots, we measured the near field component, using the hairpin antenna, near the edge of a slot in order not to disturb the field distribution.

We made numerical calculations using the FDTD (finite-difference time-domain) method where metal is assumed to be a perfect conductor which is valid in the microwave region. The grid size of the FDTD calculation was 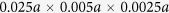.

## Additional Information

**How to cite this article**: Lee, S. and Park, Q.-H. Dynamic coupling of plasmonic resonators. *Sci. Rep.* **6**, 21989; doi: 10.1038/srep21989 (2016).

## Supplementary Material

Supplementary Information
