# Supplementary material for: Dynamic coupling of plasmonic resonators
Source: Sci Rep. 2016 Feb 25;6:21989. doi: 10.1038/srep21989 (PMC4766418; doi:10.1038/srep21989)
Supplement: Supplementary Information [file srep21989-s1.doc]

Supplemental Material:

Dynamic coupling of plasmonic resonators

Suyeon Lee, and Q-Han Park^†^

Department of Physics, Korea University, Seoul 136-701, Korea

1. **Calculation of normal modes and cross sections of coupled oscillators.**

Coupled double oscillators are described by the equations of motion,

where terms in right hand side represent the restoring force, Abraham-Lorentz force, external force, and Lorentz force between oscillators respectively. With the time harmonic dependence $x_{1}(t), x_{2}(t)\propto\exp(-i\omega t)$, and $F_{x}^{21}=q_{1}q_{2}\kappa x_{2},F_{x}^{12}=q_{1}q_{2}\kappa x_{1}$, we have

For identical oscillators, $m_{1}=m_{2}=m$, $q_{1}=q_{2}=q$, $\tau_{1}=\tau_{2}=\tau$, and $\omega_{10}=\omega_{20}=\omega_{0}$ so that

Normal modes of Eq., appearing in the absence of the external force ($E_{0}=0$), require the determinant of the matrix on the left side to vanish so that

$$\left(\omega_{0}^{2}-\omega^{2}-i\tau\omega^{3}\right)^{2}-\left(q^{2}\kappa/m\right)^{2}=0.$$

The symmetric mode with $\omega_{0}^{2}-\omega^{2}-i\tau\omega^{3}=q^{2}\kappa/m$, $x_{1}=x_{2}$ and the antisymmetric mode with $\omega_{0}^{2}-\omega^{2}-i\tau\omega^{3}=-q^{2}\kappa/m$, $x_{1}=-x_{2}$ can be selectively excited by the external force with and respectively. Steady state solutions of symmetric and antisymmetric modes are

$$\begin{matrix} x_{1}^{S}=x_{2}^{S}=\frac{qE_{0}e^{-i\omega t}/m}{\omega_{0}^{2}-\omega^{2}-i\tau\omega^{3}-q^{2}\kappa/m}, \\ x_{1}^{A}=-x_{2}^{A}=\frac{qE_{0}e^{-i\omega t}/m}{\omega_{0}^{2}-\omega^{2}-i\tau\omega^{3}+q^{2}\kappa/m}. \end{matrix}$$

The total cross section $\sigma_{c}$ of coupled oscillators, defined as the time averaged total power provided by the external field normalize by the incident intensity, is given by

.

For two normal mode of identical oscillators, total cross section becomes

where the $+(-)$ sign corresponds to the symmetric(antisymmetric) mode. Using the steady state solution, we obtain the cross section of symmetric mode $\sigma_{c}^{S}$,

$\begin{matrix} \sigma_{c}^{S}=\frac{2q^{2}\omega^{4}\left|\mathbf{x}_{1}^{S}\right|^{2}}{6\pi\varepsilon_{0}^{2}c^{4}E_{0}^{2}}+\frac{2q^{2}\omega\left|\mathbf{x}_{1}^{S}\right|^{2}\mathrm{Im}\left(\kappa\right)}{\varepsilon_{0}cE_{0}^{2}} \\ =\left\{\frac{2q^{2}\omega^{4}}{6\pi\varepsilon_{0}^{2}c^{4}E_{0}^{2}}+\frac{2q^{2}\omega\mathrm{Im}\left(\kappa\right)}{\varepsilon_{0}cE_{0}^{2}}\right\}\left|\frac{qE_{0}e^{-i\omega t}/m}{\omega_{0}^{2}-\omega^{2}-i\tau\omega^{3}-q^{2}\kappa/m}\right|^{2} \\ =\frac{q^{4}}{m^{2}}\left\{\frac{2\omega^{4}}{6\pi\varepsilon_{0}^{2}c^{4}}+\frac{2\omega\mathrm{Im}\left(\kappa\right)}{\varepsilon_{0}c}\right\}\frac{1}{\left\{\omega_{0}^{2}-\omega^{2}-q^{2}\mathrm{Re}\left(\kappa\right)/m\right\}^{2}+\left\{\tau\omega^{3}+q^{2}\mathrm{Im}\left(\kappa\right)/m\right\}^{2}}. \end{matrix}$ Also using the relation $\tau=q^{2}/6\pi\varepsilon_{0}mc^{3}$, we have

$$\begin{matrix} \sigma_{c}^{S}=\left\{\frac{2\omega^{4}}{6\pi\varepsilon_{0}^{2}c^{4}}+\frac{2\omega\mathrm{Im}\left(\kappa\right)}{\varepsilon_{0}c}\right\}\frac{\left(6\pi\varepsilon_{0}c^{3}\tau\right)^{2}}{\left\{\omega_{0}^{2}-\omega^{2}-6\pi\varepsilon_{0}c^{3}\tau\mathrm{Re}\left(\kappa\right)\right\}^{2}+\left\{\tau\omega^{3}+6\pi\varepsilon_{0}c^{3}\tau\mathrm{Im}\left(\kappa\right)\right\}^{2}} \\ =\frac{12\pi c^{2}\tau^{2}\left\{\omega^{4}+6\pi\varepsilon_{0}c^{3}\omega\mathrm{Im}\left(\kappa\right)\right\}}{\left\{\omega_{0}^{2}-\omega^{2}-6\pi\varepsilon_{0}c^{3}\tau\mathrm{Re}\left(\kappa\right)\right\}^{2}+\tau^{2}\left\{\omega^{3}+6\pi\varepsilon_{0}c^{3}\mathrm{Im}\left(\kappa\right)\right\}^{2}}. \end{matrix}$$

Similarly, we find the total cross section for the anti-symmetric mode,

$$\begin{matrix} \sigma_{c}^{A}=\frac{2q^{2}\omega^{4}\left|\mathbf{x}_{1}^{A}\right|^{2}}{6\pi\varepsilon_{0}^{2}c^{4}E_{0}^{2}}-\frac{2q^{2}\omega\left|\mathbf{x}_{1}^{A}\right|^{2}\mathrm{Im}\left(\kappa\right)}{\varepsilon_{0}cE_{0}^{2}} \\ =\frac{q^{4}}{m^{2}}\left\{\frac{2\omega^{4}}{6\pi\varepsilon_{0}^{2}c^{4}}-\frac{2\omega\mathrm{Im}\left(\kappa\right)}{\varepsilon_{0}c}\right\}\frac{1}{\left\{\omega_{0}^{2}-\omega^{2}+q^{2}\mathrm{Re}\left(\kappa\right)/m\right\}^{2}+\left\{\tau\omega^{3}-q^{2}\mathrm{Im}\left(\kappa\right)/m\right\}^{2}} \\ =\frac{12\pi c^{2}\tau^{2}\left\{\omega^{4}-6\pi\varepsilon_{0}c^{3}\omega\mathrm{Im}\left(\kappa\right)\right\}}{\left\{\omega_{0}^{2}-\omega^{2}+6\pi\varepsilon_{0}c^{3}\tau\mathrm{Re}\left(\kappa\right)\right\}^{2}+\tau^{2}\left\{\omega^{3}-6\pi\varepsilon_{0}c^{3}\mathrm{Im}\left(\kappa\right)\right\}^{2}}. \end{matrix}$$

Figure S1 shows the spectrum of scattering cross section of anti-symmetric mode varying the separation similar to Fig. 2 for the symmetric case. The vertical cut in (b) represents the scattering spectra at three different separations. The horizontal cut in (c) shows the increasing behaviour of cross section for decreasing separation unlike the symmetric mode case.


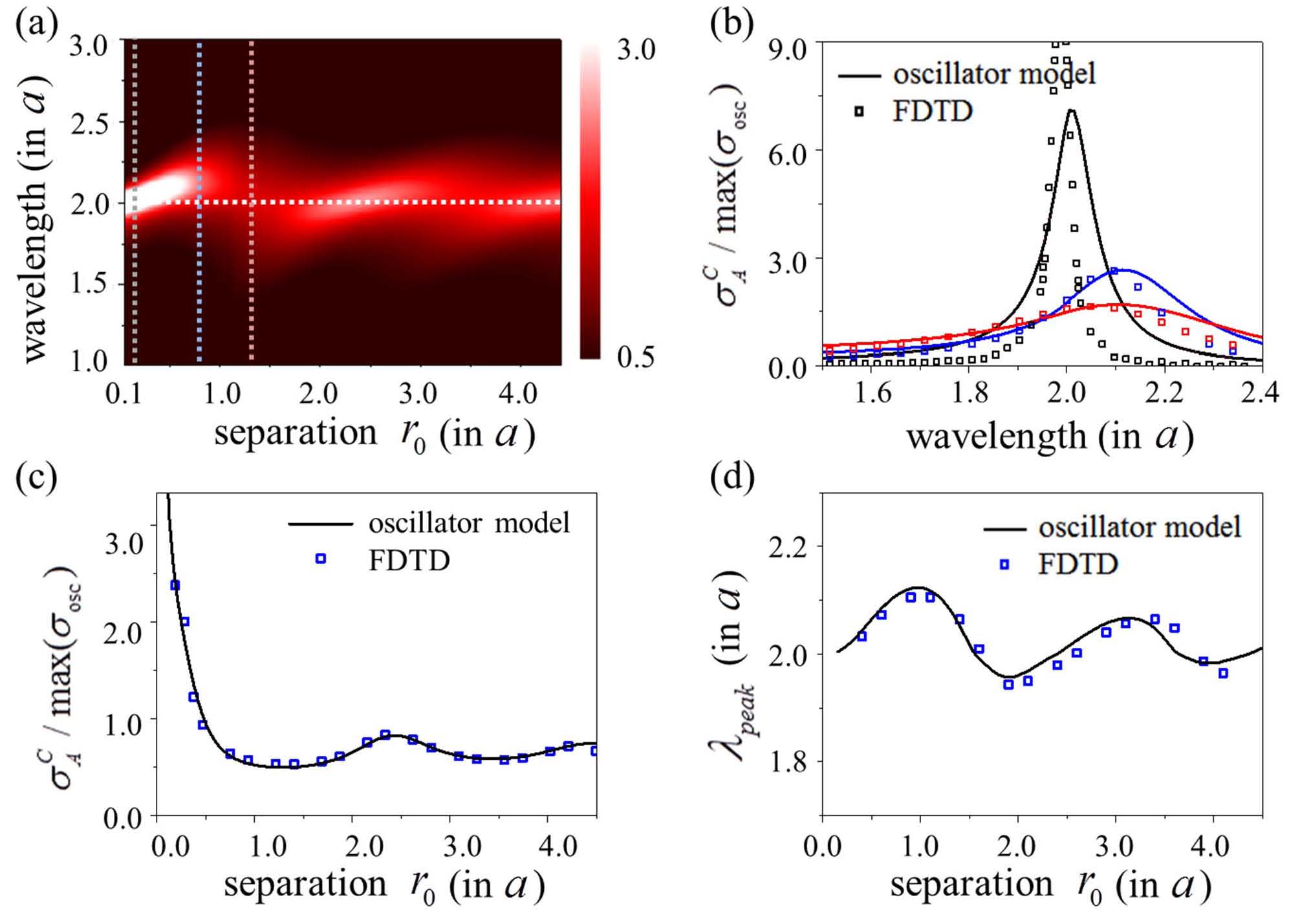


**Figure S1**. (a) Scattering cross section of the anti-symmetric mode calculated from the oscillator model varying incident wavelength and separation. Scattering cross section is normalized by the cross section of resonant single oscillator at the resonance, and wavelength and separation are in the unit of the long side of the rectangle. (b) Vertical breaks of (a) from the oscillator model (line) in comparison with the FDTD results (square dot). Separations between oscillators are $0.2a$ (black), $0.8a$ (blue), and $1.3a$ (red). (c) Horizontal break of (a) at resonance wavelength (black line) and the FDTD result (square dot). (d) Resonance peak shifts of the cross section in anti-symmetric mode.

1. **Derivation of the dynamic coupling constant**

The electric and magnetic fields $\mathbf{E}_{21}$ and $\mathbf{B}_{21}$ generated by charge $q_{2}$ and measured at retarded time $t-r/c$are given by

where $\mathbf{r}=\mathbf{r}_{1}-\mathbf{r}_{2}=r\hat{r}$ with the unit vector $\hat{r}$. $\mathbf{r}_{1}$ and $\mathbf{r}_{2}$ are position vectors of charge $q_{1}$ and $q_{2}$ respectively. The subscript “ret” indicates that all quantities are evaluated at retarded time $t-r/c$. The Lorentz force $\mathbf{F}_{21}$ acting on $q_{1}$ is . We assume that charge moves along the x-direction so that only x-component of Lorentz force $F_{x}^{21}$ is of interest. For non-relativistic region, ,

We also assume that displacements $x_{1}\left(t\right)$ and $x_{2}\left(t\right)$ are much smaller than the separation $d$ so that $\mathbf{r}\approx\left[d+\left(b_{1}+b_{2}\right)/2\right]\hat{y}\equiv r_{0}\hat{y}$ and $\mathbf{x}_{2}\left(t\right)$ is orthogonal to $\mathbf{r}$. Since only electric field contributes to the x-component of the Lorentz force, we have

Using the harmonic dependence $\mathbf{x}_{2}(t)\propto\exp(-i\omega t)$and evaluating at retarded time, we have

where

$$\kappa=\frac{S^{2}}{4\pi\varepsilon_{0}}\left[\frac{i\omega}{cr_{0}^{2}}+\frac{\omega^{2}}{c^{2}r_{0}}\right]e^{i\omega r_{0}/c}$$

1. **Determination of screening factor**

Without the screening factor, force between two oscillators diverges as $r_{0}$ goes to zero which can be seen in Eq. . Here, we determine the screening factor $S$ using the property of symmetrically coupled identical resonators. In the limit of zero separation, coupled identical slots merge into a single slot resonator so that the total cross section of coupled resonators becomes that of a single resonator. We also require the screening factor S to be 1 in the infinite separation. The ratio of cross sections between the coupled symmetric mode and the single oscillator is

$$\begin{matrix} \frac{\sigma_{c}^{S}}{\sigma_{Single}}=\frac{\left(\omega_{0}^{2}-\omega^{2}\right)^{2}+\tau^{2}\omega^{6}}{6\pi c^{2}\tau^{2}\omega^{4}}\frac{12\pi c^{2}\tau^{2}\left\{\omega^{4}+6\pi\varepsilon_{0}c^{3}\omega\mathrm{Im}\left(\kappa\right)\right\}}{\left\{\omega_{0}^{2}-\omega^{2}-6\pi\varepsilon_{0}c^{3}\tau\mathrm{Re}\left(\kappa\right)\right\}^{2}+\tau^{2}\left\{\omega^{3}+6\pi\varepsilon_{0}c^{3}\mathrm{Im}\left(\kappa\right)\right\}^{2}} \\ =\frac{\left(\omega_{0}^{2}-\omega^{2}\right)^{2}+\tau^{2}\omega^{6}}{\omega^{4}}\frac{2\left\{\omega^{4}+6\pi\varepsilon_{0}c^{3}\omega\mathrm{Im}\left(\kappa\right)\right\}}{\left\{\omega_{0}^{2}-\omega^{2}-6\pi\varepsilon_{0}c^{3}\tau\mathrm{Re}\left(\kappa\right)\right\}^{2}+\tau^{2}\left\{\omega^{3}+6\pi\varepsilon_{0}c^{3}\mathrm{Im}\left(\kappa\right)\right\}^{2}}. \end{matrix}$$

Near resonance, we have

$$\frac{\sigma_{c}^{S}}{\sigma_{Single}}\approx\frac{2\omega^{2}\left\{\omega^{4}+6\pi\varepsilon_{0}c^{3}\omega\mathrm{Im}\left(\kappa\right)\right\}}{\left\{6\pi\varepsilon_{0}c^{3}\mathrm{Re}\left(\kappa\right)\right\}^{2}+\left\{\omega^{3}+6\pi\varepsilon_{0}c^{3}\mathrm{Im}\left(\kappa\right)\right\}^{2}}.$$

Also in the zero separation limit, we note that the imaginary part of $\kappa$ is dominant so that

$$\frac{\sigma_{c}^{S}}{\sigma_{Single}}\approx\frac{2\omega^{3}}{\omega^{3}+6\pi\varepsilon_{0}c^{3}\mathrm{Im}\left(\kappa\right)},$$

Finally, the requirement of single slot limit $\sigma_{c}^{S}/\sigma_{Single}=1$ determines $\mathrm{Im}\left(\kappa\right)\approx\omega^{3}/6\pi\varepsilon_{0}c^{3}$ and the screening factor

$$S=1-\exp\left(-\sqrt{2/3}\omega r_{0}/c\right).$$

1. **EIT spectrum**

The steady state solution of coupled non-identical oscillators is

$$\begin{matrix} x_{1}=\frac{q_{1}}{m_{1}}\frac{\left[\omega_{20}^{2}-\omega^{2}-i\tau_{2}\omega^{3}+6\pi\varepsilon_{0}c^{3}\tau_{2}\kappa\right]E_{0}e^{-i\omega t}}{\left(\omega_{10}^{2}-\omega^{2}-i\tau_{1}\omega^{3}\right)\left(\omega_{20}^{2}-\omega^{2}-i\tau_{2}\omega^{3}\right)-(6\pi\varepsilon_{0}c^{3})^{2}\tau_{1}\tau_{2}\kappa^{2}}, \\ x_{2}=\frac{q_{2}}{m_{2}}\frac{\left[\omega_{10}^{2}-\omega^{2}-i\tau_{1}\omega^{3}+6\pi\varepsilon_{0}c^{3}\tau_{1}\kappa\right]E_{0}e^{-i\omega t}}{\left(\omega_{10}^{2}-\omega^{2}-i\tau_{1}\omega^{3}\right)\left(\omega_{20}^{2}-\omega^{2}-i\tau_{2}\omega^{3}\right)-(6\pi\varepsilon_{0}c^{3})^{2}\tau_{1}\tau_{2}\kappa^{2}}. \end{matrix}$$

For coupled oscillators corresponding to the tightly coupled resonators, the dynamic coupling is pure imaginary, $\kappa\approx i\omega^{3}/6\pi\varepsilon_{0}c^{3}$. As a result, Eq. is approximated as

$$\begin{matrix} x_{1}\approx\frac{q_{1}}{m_{1}}\frac{\left(\omega_{20}^{2}-\omega^{2}\right)E_{0}e^{-i\omega t}}{\left(\omega_{10}^{2}-\omega^{2}-i\tau_{1}\omega^{3}\right)\left(\omega_{20}^{2}-\omega^{2}-i\tau_{2}\omega^{3}\right)+\tau_{1}\tau_{2}\omega^{6}}, \\ x_{2}\approx\frac{q_{2}}{m_{2}}\frac{\left(\omega_{10}^{2}-\omega^{2}\right)E_{0}e^{-i\omega t}}{\left(\omega_{10}^{2}-\omega^{2}-i\tau_{1}\omega^{3}\right)\left(\omega_{20}^{2}-\omega^{2}-i\tau_{2}\omega^{3}\right)+\tau_{1}\tau_{2}\omega^{6}}. \end{matrix}$$

And the total corresponding cross section becomes

.
